# A proof-of-concept study of extracting patient histories for rare/intractable diseases from social media

**DOI:** 10.5808/GI.2020.18.2.e17

**Published:** 2020-06-18

**Authors:** Atsuko Yamaguchi, Núria Queralt-Rosinach

**Affiliations:** 1 Tokyo City University, Setagaya, Tokyo 157-0087, Japan; 2 Leiden University Medical Center, Leiden, 2333 ZA, The Netherlands

**Keywords:** intractable diseases, rare diseases, social media mining

## Abstract

The amount of content on social media platforms such as Twitter is expanding rapidly. Simultaneously, the lack of patient information seriously hinders the diagnosis and treatment of rare/intractable diseases. However, these patient communities are especially active on social media. Data from social media could serve as a source of patient-centric knowledge for these diseases complementary to the information collected in clinical settings and patient registries, and may also have potential for research use. To explore this question, we attempted to extract patient-centric knowledge from social media as a task for the 3-day Biomedical Linked Annotation Hackathon 6 (BLAH6). We selected amyotrophic lateral sclerosis and multiple sclerosis as use cases of rare and intractable diseases, respectively, and we extracted patient histories related to these health conditions from Twitter. Four diagnosed patients for each disease were selected. From the user timelines of these eight patients, we extracted tweets that might be related to health conditions. Based on our experiment, we show that our approach has considerable potential, although we identified problems that should be addressed in future attempts to mine information about rare/intractable diseases from Twitter.

**Availability:** In this paper, we used Twitter timelines and the Human Phenotype Ontology. We obtained user timelines from Twitter (https://twitter.com) using Python code (https://github.com/acopom/smm4rd) with Tweepy (https://www.tweepy.org/), which is a Python library for accessing the Twitter API (https://developer.twitter.com/). The Human Phenotype Ontology is available at https://hpo.jax.org/app/download/ontology.

## Introduction

Social media has become a data source that is making a major contribution to big data. Recent scientific research has started to use and evaluate social media in the context of healthcare [1-4]. Svenstrup et al. [5] highlighted the potential of social media platforms dedicated to healthcare specialists as a means of knowledge-sharing for rare disease (RD) diagnoses. Schumacher et al. [6] introduced a case of online research and analysis of respondents using social media for the study of RDs. The role of social media was as a “participation caption” for recruiting a patient cohort and collecting clinical information. The authors concluded that the methodology and response patterns can be used for RD research. However, in those studies, social media platforms were used only from the viewpoint of healthcare specialists (e.g., medical doctors), even though a much broader range of people, including patients, are contributing to social media data. In particular, communities of patients suffering from RDs are very active on social media platforms. By definition, RDs affect small percentages of the population (https://ec.europa.eu/info/research-and-innovation/research-area/health-research-and-innovation/rare-diseases_en). These RD patient communities are small and patients are geographically scattered. Even though there are more than 8,000 RDs, only 5% have treatment. The lack of patient information available for research seriously hinders the diagnosis and treatment of rare and intractable diseases [7]. In general, RD patients suffer from very severe and heterogeneous symptoms and remain undiagnosed for several years [8]. Consequently, these disease communities use social media platforms to try to find other patients with similar health problems or expertise about their rare condition, sharing manifold types of information—including symptoms, treatments, side effects, and other diseases and activities—that go beyond what is normally captured in a clinical setting or patient registry [9]. Recently, Klein et al. [10] mined Twitter to collect data on rare health-related events reported by patients, and showed that this social media platform was useful for gathering patient-centric information that could be used for future epidemiological analyses. Our hypothesis was that data from RD patient histories posted on social media would capture patients’ perspectives of their health status, which may be valuable for research into ways of helping undiagnosed patients by accelerating the timeline to diagnosis and treatment.

The special theme of the Biomedical Linked Annotation Hackathon 6 (BLAH6) was “social media mining.” Therefore, we attempted to extract patient-centric knowledge from social media as a task for the 3-day hackathon. In this paper, we present our work that we conceived, designed, and developed during BLAH6 to explore the potential of social media data as a source of patient-centric knowledge. For this project, we focused on rare and intractable diseases and selected Twitter to obtain patients’ timelines, as this platform may contain descriptions of the history of their health conditions. By focusing on the date of diagnosis, we intended to obtain histories of their health conditions before and after diagnosis.

## Methods

Due to the time constraints of the hackathon, we selected one RD and one intractable disease. Then, we searched for patients with the two diseases and obtained their timelines. We also tried to extract tweets related to the disease and symptoms from each timeline.

First, we selected a RD that is adult-onset and not too rare to facilitate the extraction of a proper amount of data for analysis. To do so, we used information on the number of patients diagnosed with rare and intractable diseases in Japan, provided by Japan Intractable Disease Information Center (https://www.nanbyou.or.jp/). Based on this information, we selected amyotrophic lateral sclerosis (ALS) as an RD, and for similar reasons, we selected multiple sclerosis (MS) as an intractable disease. Second, we obtained a list of Twitter users who were diagnosed with ALS or MS using the search terms “I was diagnosed” and the disease name. Then, we selected users diagnosed during the last 5 years who had more than 100 tweets, excluding retweets and replies. This resulted in four users for each disease. By using Tweepy with a Python script (https://github.com/acopom/smm4rd), we obtained the timestamp and the text of the Twitter timelines, including 6088 tweets without retweets and replies for the eight users.

To extract tweets dealing with a user’s health conditions, we used all terms in the Human Phenotype Ontology (HPO) [11] except for three (“all,” “left,” and “right”). All tweets that included HPO terms in the text were extracted. We then removed some tweets by manual search inspection because they described the health condition of someone else, such as the user’s child. Through this process, we obtained a set of tweets that were related to the user’s health condition. We called this set of tweets “tweets by HPO” for a user u and denoted it as *H(u)*.

Additionally, we extracted tweets dealing with health conditions using common words, such as “cold.” However, many tweets extracted in this way were not related to health conditions, for example, “It’s cold today.” Consequently, we manually removed many tweets from this extracted tweet set. We called this set of tweets “tweets by manual” for a user u and denoted it as *M(u)*.

We called *H(u)* ∪ *M(u)* “tweets about the disease” and denoted this set as *D(u)*. As each tweet in *D(u)* may contain sensitive information from the viewpoint of user protection, a short summary of each tweet to conceal details was made manually.

## Results and Discussion

To conceal the identity of the users with ALS and MS, we used ALS1, ALS2, ALS3, and ALS4 to refer to the ALS patients and MS1, MS2, MS3, and MS4 to refer to the MS patients instead of their Twitter user names. Table 1 shows the numbers of tweets, the number of tweets in *H(u)*, and the number of tweets in *M(u)* for each user *u*. Of note, all tweets about ALS were posted after the users were diagnosed, whereas all tweets about MS, except for one, were posted before the diagnosis.

**Fig. 1.**
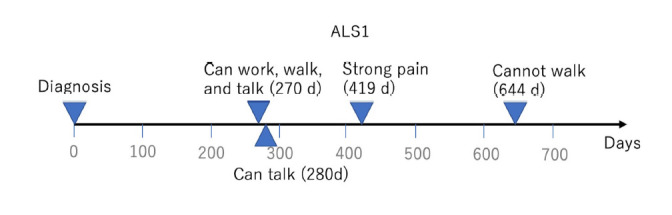
Patient history with four events constructed by five tweets in *D*(ALS1). ALS1, amyotrophic lateral sclerosis 1.

We next constructed a patient history for each user *u* using tweets in *D(u)*. For example, ALS1 had two tweets in *H*(ALS1) extracted by the HPO term “pain” (HP:0012531). *M*(ALS1) included three tweets that were extracted manually. From these five tweets, we obtained four events related to health conditions because two of the tweets in *H*(ALS1) indicated one event. Fig. 1 shows the patient history of ALS1, who had four events after diagnosis. We set the date of diagnosis as a reference point. We presented short summaries such as “can talk” instead of showing real tweets because the extracted tweets may contain sensitive information from the viewpoint of user protection. At 270 days after the date of diagnosis, we can see that ALS1 could work, walk, and talk. However, ALS1 could no longer walk 644 days after the date of diagnosis.

**Fig. 2.**
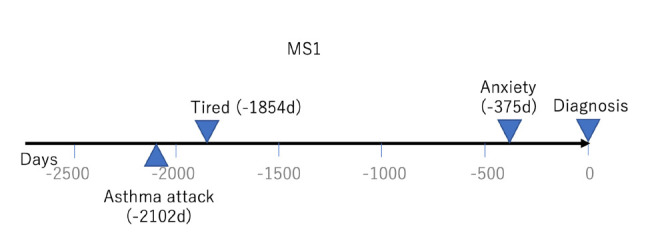
Patient history with three events constructed by four tweets in *D*(MS1). MS, multiple sclerosis.

Similarly, Fig. 2 shows the patient history of MS1, who had three events as constructed by four tweets in *D*(MS1). MS1 had an asthma attack 2,102 days before the diagnosis, and experienced anxiety and received a drug for it 375 days before the diagnosis.

This experiment showed the potential of Twitter data as a source of patient-centric knowledge, by extracting tweets related to health conditions and constructing a patient history from each user’s timeline. However, we found that the typical method of scientific data extraction did not work well for mining tweets. As shown in Table 1, we obtained a very small number of tweets related to health conditions. To address this limitation, the development of a dictionary for the healthcare domain specialized for social media data is vitally necessary to leverage and better understand the scientific value of data from social media for rare and intractable diseases.

## Figures and Tables

**Table 1.** Summary of the eight users analyzed in this experiment

User	#Tweets	#*H*	#*M*
ALS1	2135	2	3
ALS2	1295	0	0
ALS3	213	1	1
ALS4	182	7	5
MS1	777	3	1
MS2	348	1	0
MS3	572	0	2
MS4	566	2	1
Total	6088	16	13

#Tweets, #H, and #M show the total numbers of tweets, the number of tweets in H, and the number of tweets in M, respectively.
